# Supplementary figures and images for: Exosomal miRNA Profile in Small-for-Gestational-Age Children: A Potential Biomarker for Catch-Up Growth
Source: Genes (Basel). 2022 May 24;13(6):938. doi: 10.3390/genes13060938 (PMC9223036; doi:10.3390/genes13060938)

## Slide 1
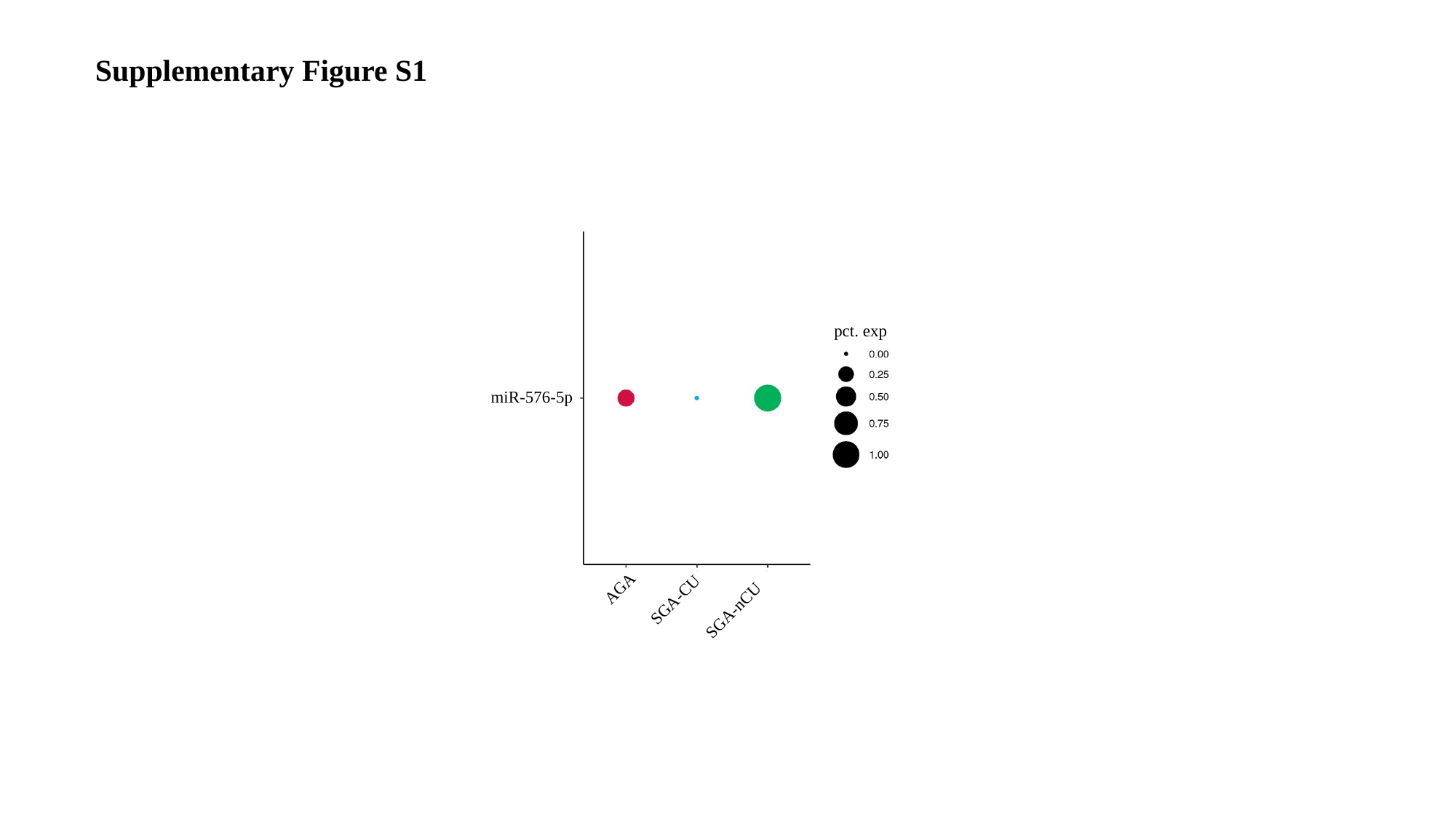

Supplementary Figure S1
miR-576-5p
AGA
SGA-CU
SGA-nCU
pct. exp

Supplement: Supplementary file 1 [file genes-13-00938-s001.zip › genes-1741600-supplementary.pptx]
